# Expression of *Hox* Genes in Early Development of Irregular Sea Urchin *Scaphechinus mirabilis* (Echinodermata, Echinoidea)

**DOI:** 10.1134/S0012496625600526

**Published:** 2025-12-02

**Authors:** O. V. Ezhova, N. V. Ageenko, K. V. Kiselev, V. V. Malakhov

**Affiliations:** 1 Biological Faculty, Moscow State University, Moscow, Russia; 2 A.V. Zhirmunsky National Scientific Center of Marine Biology, Far East Branch, Russian Academy of Sciences (NSCMB FEB RAS), Vladivostok, Russia; 3 Federal Scientific Center of the East Asian Terrestrial Biodiversity, Far East Branch, Russian Academy of Sciences, Vladivostok, Russia

**Keywords:** blastula, gastrula, pluteus, prism stage, real-time, Regularia, sand dollars

## Abstract

Current data indicate that the *Hox* gene expression pattern differs between regular and irregular sea urchins. Expression of most genes of the *Hox* cluster is not activated until the pluteus forms, and only *Hox7*, *11*/*13b*, and *11*/*13c* are expressed at the blastula stage in two Regularia species examined previously, *Strongylocentrotus purpuratus* and *St. intermedius*. In contrast, in *Peronella japonica*, the only species studied in Irregularia, *Hox7*, *9*/*10*, *11*/*13b*, and *11*/*13c* are expressed at the blastula and gastrula stages; *Hox1*, *7, 8, 9*/*10, 11*/*13a*, and *11*/*13b* are expressed at the prism stage; and most genes of the *Hox* cluster are activated at the pluteus stage. The irregular sea urchin *Scaphechinus mirabilis* was examined. Almost all of its *Hox* genes were silent at early stages. *SmHox11*/*13b* was an exception and was expressed at the gastrula stage. Most *Hox* genes were activated only at the pluteus stage. Thus, the expression pattern of the *Hox* cluster in *Sc. mirabilis* is similar to that of the regular sea urchins *St. purpuratus* and *St. intermedius* and differs from that of the other irregular sea urchin *P. japonica*.

Echinodermata is a phylum of marine invertebrates that undergo substantial rearrangements of the body plan during their development. The rearrangements reflect the intricate evolutionary history of the phylum. Planktonic larvae formed in echinoderms during ontogeny are bilaterally symmetrical, but display dissymmetry in the structure of coelomic derivatives [1]. A complex rearrangement of the organization occurs in larvae of Echinodermata during metamorphosis, resulting in secondary radial symmetry, which is typical for echinoderms. Genes of the *Hox* family are known to play a crucial role in regulating the layout of organs along the anteroposterior axis in Triploblastica, and spatial and temporal collinearity is characteristic of their expression [2, 3]. However, dramatic deviations of spatial and temporal collinearity in *Hox* gene expression have been observed in echinoderms. For example, the central and posterior gene groups of the *Hox* cluster are the first to be expressed in Echinodermata species examined in this respect, while the anterior group is expressed first in most Triploblastica [4–9]. Distortions in the order of expression of the regulatory genes might be associated with intricate morphological rearrangements, which have occurred in members of the phylum Echinodermata in the course of their evolution [10].

Two main body plans are observed in sea urchins (Echinoidea). Species of the so-called regular sea urchins (the subclass Regularia) show pentameric radial symmetry in adults, as typical in echinoderms. Irregular sea urchins (the subclass Irregularia) are characterized by secondary bilateral symmetry, which develops on the basis of radial symmetry and has a plane that does not coincide with the plane of bilateral symmetry of Triploblastica [11]. Expression of genes of the *Hox* cluster during development has been studied in two regular sea urchin species of the family Strongylocentrotidae, *Strongylocentrotus purpuratus* (Stimpson, 1857) and *St. intermedius* (A. Agassiz, 1864), and the following regularities have been observed. Most of the *Hox* genes remain inactive until a well-developed planktotrophic pluteus larva forms. The *Hox7*, *11*/*13b,* and *11*/*13c* genes are the only exception, being expressed at the blastula stage [4, 9, 12]. Another *Hox* gene expression pattern has been observed in the irregular sea urchin *Peronella japonica* Mortensen, 1948, which belongs to the family Laganidae (Table 1). *PjHox7*, *9*/*10*, *11*/*13b,* and *11*/*13c* are expressed at the blastula and gastrula stages; *PjHox1*, *8,* and *11*/*13a* come to be expressed along with the above genes at the prism stage; and all but one gene of the *Hox* cluster are expressed in the pluteus larva 48 h post-fertilization (hpf), with the exception of silenced *PjHox11*/*13c* [7]. It is therefore possible to assume that activity of the *Hox* cluster genes in early development differs between regular and irregular sea urchins. To check the assumption, we qualitatively assessed early expression of all of the 11 *Hox* genes in the developing irregular sea urchin *Scaphechinus mirabilis* A. Agassiz, 1864, which belongs to the family Scutellidae.

**Table 1.** Expression pattern of the *Hox* genes during early development in the sea urchin species examined to date

Subclass	Species	Developmental stage					Source
		Blastula	Gastrula	Prism stage	Early pluteus	Late pluteus	
Regularia	*Strongylocentrotus purpuratus*	**	**	**	**	********	[4]
	*St. intermedius*	***	–	*	***********	*********	[9]
Irregularia	*Peronella japonica*	***	****	******	*******	*********	[7]
	*Scaphechinus mirabilis*	–	*	–	****	**	our data

Asterisks show the number of genes with detectable expression in the *Hox* cluster; (–), expression undetectable.

The following *Sc. mirabilis* developmental stages were examined: blastula, 13 hpf; gastrula, 35 hpf; prism stage, 46 hpf; early pluteus, 4 days post-fertilization (dpf); and late pluteus, 9 dpf (Fig. 1). All experiments with sea urchin fertilization and development were carried out at the Vostok Marine Biological Station (Zhirmunsky National Scientific Center of Marine Biology, Far East Branch, Russian Academy of Sciences (NSCMB FEB RAS)). Urchins were collected in the Vostok Bay, Sea of Japan. The urchins were kept in tanks with running aerated water until use and were washed with UV-irradiated filtered seawater immediately before experiments. Spawning was induced by injecting 1–2 mL of 0.5 M potassium chloride. Larvae were obtained via artificial fertilization and cultured at 18°C.

**Fig. 1.**
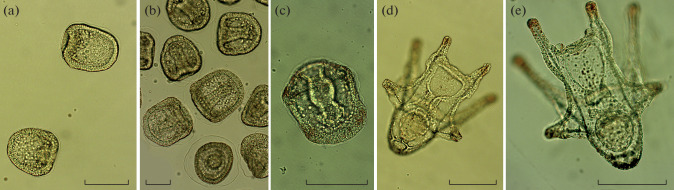
*Scaphechinus mirabilis* developmental stages examined in this study: (a) blastula, 13 hpf; (b) gastrula, 35 hpf; (c) prism stage, 46 hpf; (d) early pluteus, 4 dpf; and (e) late pluteus, 9 dpf. Bar, 50 μm.

Total RNA was isolated from eggs and larvae as described by Kiselev et al. [13]. The total RNA amount was assessed by spectrophotometry (NanoPhotometer P-300, IMPLEN, Germany).

Complementary DNA (cDNA) was synthesized using a reverse transcription kit (Evrogen, Russia) and 2 µg of total RNA. The reaction mixture (50 µL) for reverse transcription–polymerase chain reaction (RT–PCR) contained a 1× RT buffer, 0.24 mM each deoxynucleotide triphosphate (dNTP), 0.2 μM oligo-(dT)_15_ primer, and 200 units of MMLV reverse transcriptase. The reaction was carried out at 37°C for 2 h. The reaction products (0.5 µL) were amplified via real-time PCR (qPCR).

To theoretically design the qPCR primers directed to the *Sc. mirabilis* genes, we analyzed the nucleotide sequence of the *St. purpuratus Hox* gene cluster [14, 15]. All data and respective query and visualization tools are available via the SpBase public database of sea urchin genomic sequences (http://www.spbase.org/SpBase/ rnaseq/) and the NCBI bioproject database (http://www. ncbi.nlm.nih.gov/bioproject), acc. no. PRJNA81157. NCBI Primer Blast software were used to design the primers. To check the specificity of the resulting primers, PCR products were sequenced (ABI 3130 Genetic Analyser, Applied Biosystems, United States). Sequencing was carried out at the Federal Research Center of East Asian Terrestrial Biodiversity. PCR product sequencing was repeated at least three times for each time point, and all of the resulting sequences flanked by the primers (150–200 bp) did not substantially differ from the respective *St. purpuratus* gene sequences. To normalize the results obtained for *Sc. mirabilis,* the actin (GenBank acc. no. DQ229162) and ubiquitin (GenBank acc. no. LOC754856) gene sequences were used as an endogenous control. Data were collected in five independent experiments. Primers used to amplify the actin and ubiquitin genes in qPCR were as described previously [16].

The expression level was estimated in relative (rel.) units for each individual *Sc. mirabilis Hox* gene. The transcription level of a target gene in unfertilized eggs was taken to be unity. Quantitative data were thus obtained to characterize the expression levels of all of the 11 *Sc. mirabilis* *Hox* genes at early developmental stages.

Expression levels of most genes of the *Sc. mirabilis Hox* gene cluster was extremely low in the first hours and days of development and did not exceed the respective expression levels in unfertilized eggs (Fig. 2). Within the first two days post-fertilization, *SmHox11*/*13b* was the only *Hox* gene expressed to a level higher than unity at the gastrula stage (35 hpf). The level was a maximum observed for the gene across all stages examined. An overall increase in expression of all but one (*SmHox11*/*13b*) *Hox* gene was detected in 4-day-old pluteus larvae. However, only the *SmHox8* and *SmHox11*/*13c* genes showed expression levels higher than unity at that stage. Expression of most *Hox* genes decreased again in 9-day-old pluteus larvae, with the exception of *SmHox6*, *SmHox9*/*10* (expression levels higher than unity), and *SmHox11*/*13b.* However, the expression level of the last gene at that stage did not reach the level observed at the gastrula stage (Fig. 2).

**Fig. 2.**
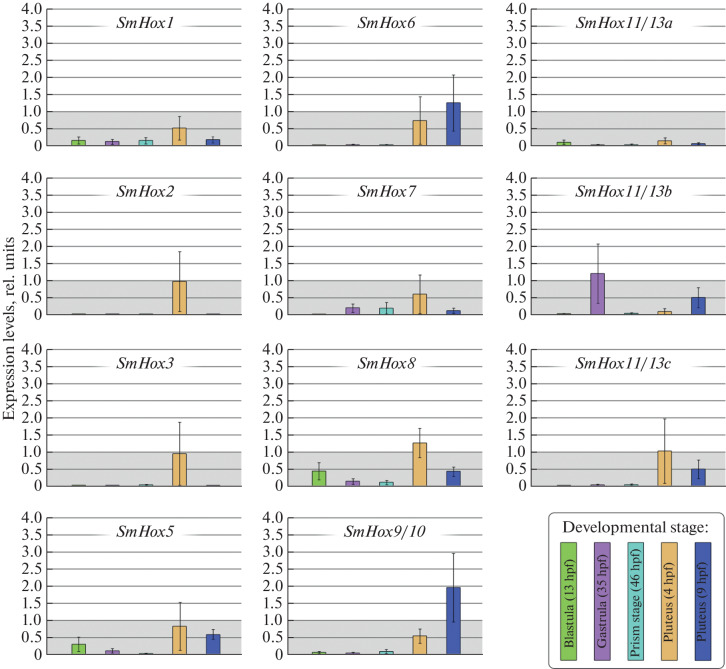
Quantitative assessment of the expression levels of the *Hox* cluster genes in *Sc. mirabilis* at early developmental stages. The levels were measured in rel. units, taking the amplification level of the respective gene in unfertilized eggs to be unity.

Several similarities in quantitative *Hox* gene expression patterns at early developmental stages were observed between the irregular sea urchin *Sc. mirabilis* and the regular sea urchin *St. intermedius* [9]. An overall increase in expression of all *Hox* genes in 4-day-old pluteus larvae is similarly characteristic of the *St. intermedius* development. In the first two days, the *SiHox11*/*13b* gene is similarly expressed to the highest level, but at the blastula, rather than gastrula, stage. In addition, the *SiHox2*, *3*, *5*, *8,* and *9*/*10* genes are similar in expression dynamics to *SmHox2*, *3*, *5*, *8*, *9/10* (the pattern of Hox8 expression differedon  differed  only  in 9-day-old pluteus larvae). In the other regular sea urchin *St. purpuratus* [4], expression of *SpHox11*/*13b* similarly increases to a considerable extent at the mesenchyme blastula stage (21–36 hpf), decreases abruptly at the late gastrula stage (48 hpf), and gradually increases again at the pluteus stage. A similar expression pattern has been observed for *SpHox7*, but without an abrupt decrease in expression at the gastrula stage [4]. In the irregular sea urchin *P. japonica* [7], activation of *PjHox7*, *11*/*13b,* and *11/13c* occurs as early as the blastula stage (6–10.5 hpf). *PjHox9*/*10* comes to be expressed along with the above genes at the gastrula stage (13–15 hpf), and expression of *PjHox1*, *8*, and *11*/*13a* is added at the prism stage (18–21 hpf), while *PjHox11*/*13c* expression has no longer been detected to the end of the experiment (up to the 3-day-old pluteus stage). Almost all *Hox* genes are already expressed in 2-day-old pluteus larvae in *P. japonica*, with the exception of *PjHox11*/*13c*, which is expressed only in the blastula and early gastrula, and *PjHox2*, whose expression has not been detected throughout the experiment [7]. In *Sc. mirabilis*, which was examined in this work, expression levels exceeding unity were observed for *SmHox8* (in 4-day-old pluteus larvae) and *SmHox9*/*10* (in 9-day-old pluteus larvae) (Fig. 2). The expression pattern of *PjHox11*/*13c* with gene activation at the earliest developmental stages and subsequent lack of expression at the prism and pluteus stages resembled that of *SmHox11*/*13b* to a greater extent.

In total, the expression pattern of the genes belonging to the *Hox* cluster in the irregular sea urchin *Sc. mirabilis* is similar to that in the regular sea urchins *St. purpuratus* and *St. intermedius* and differs from that in the other irregular sea urchin *P. japonica* (Table 1). It seems that differences in the expression pattern of the *Hox* cluster genes are not determined by whether a species belongs to Regularia or Irregularia, but depend on other, still unidentified factors.
